# Tuning the chemiluminescence of a luminol flow using plasmonic nanoparticles

**DOI:** 10.1038/lsa.2016.164

**Published:** 2016-11-04

**Authors:** Alina Karabchevsky, Ali Mosayyebi, Alexey V Kavokin

**Affiliations:** 1 Electrooptical Engineering Unit and Ilse Katz Institute for Nanoscale Science & Technology, Ben-Gurion University, Beer-Sheva 84105, Israel; 2 Engineering Sciences Unit, Engineering and the Environment, University of Southampton, Southampton SO17 1BJ, UK; 3 Department of Physics and Astronomy, University of Southampton, Southampton SO17 1BJ, UK; 4 CNR-SPIN, Viale del Politecnico 1, I-00133 Rome, Italy

**Keywords:** chemiluminescence, microfluidics, nanoparticles, plasmonics

## Abstract

We have discovered a strong increase in the intensity of the chemiluminescence of a luminol flow and a dramatic modification of its spectral shape in the presence of metallic nanoparticles. We observed that pumping gold and silver nanoparticles into a microfluidic device fabricated in polydimethylsiloxane prolongs the glow time of luminol. We have demonstrated that the intensity of chemiluminescence in the presence of nanospheres depends on the position along the microfluidic serpentine channel. We show that the enhancement factors can be controlled by the nanoparticle size and material. Spectrally, the emission peak of luminol overlaps with the absorption band of the nanospheres, which maximizes the effect of confined plasmons on the optical density of states in the vicinity of the luminol emission peak. These observations, interpreted in terms of the Purcell effect mediated by nano-plasmons, form an essential step toward the development of microfluidic chips with gain media. Practical implementation of the discovered effect will include improving the detection limits of chemiluminescence for forensic science, research in biology and chemistry, and a number of commercial applications.

## Introduction

**Figure 1:**
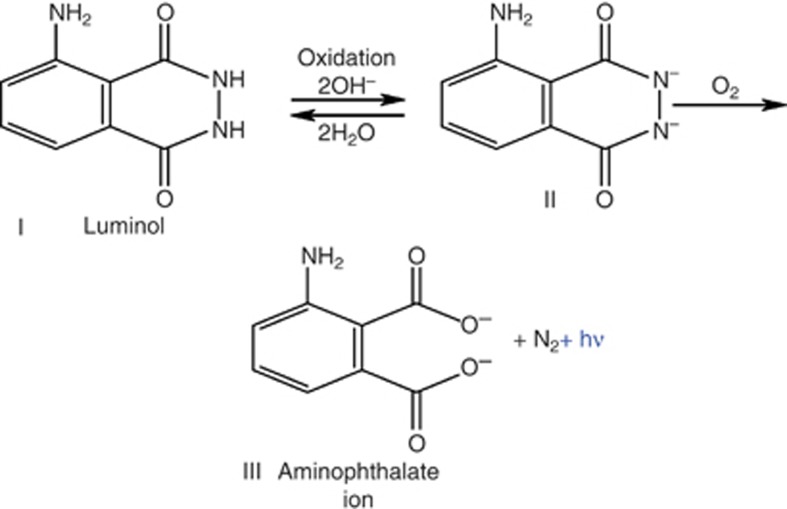
The chemiluminescence reaction of luminol C_8_H_7_N_3_O_2_ (I) in relatively nonacidic solvents, resulting in aminophthalate ion (III), which is a light-emitting species^2^.

Chemiluminescence is a fascinating optical effect that is used in various applications, from forensic science to industrial biochemistry. Luminol is a chemical that exhibits chemiluminescence (Figure 1), emitting a blue glow. Approximately five decades ago, luminol was used for the first time to analyze a crime scene in Germany^1^. Since then, it has become a very popular criminology tool, as it can reveal blood stains. A mixture of luminol, hydrogen peroxide and a thickening agent can be sprayed on surfaces contaminated with blood traces. If catalyzed by metal ions, such as the iron contained in blood hemoglobin, the mixture will glow.

Criminologists use luminol to identify microscopic blood drops invisible to the naked eye. Luminol is widely used in biological and chemical research as a marker for iron, for determination of benzoyl peroxide in flour and more^3, 4, 5, 6^. It also helps to detect low concentrations of hydrogen peroxide, proteins and DNA. Several methods have been proposed for the specific, sensitive and amplified detection of DNA utilizing chemiluminescence. Among them is a rapidly progressing method to image biosensing events on surfaces, termed as electrogenerated chemiluminescence^7^. The primary advantage of chemiluminescence compared to the widely used fluorescence^8, 9^ is the generation of photons during the course of a chemical reaction. In this case, the detected signal is not affected by external light scattering, source fluctuations or high background due to nonresonant excitation^10^. Consequently, illuminometers based on light detection by photomultiplier tubes are among the cheapest devices in the field^11^.

Here, we study luminol flows in a microfluidic device. Microfluidic devices reduce liquid consumption, provide well-controlled mixing and particle manipulation, integrate and automate multiple assays (known as lab-on-a-chip), and facilitate imaging and tracking^12^. The continuous flow injection provides improved mixing between the luminol and oxidant, resulting in a higher intensity of emitted light than in a cuvette. The typical glow time when luminol is in contact with an activating oxidant is only ~30 s. However, flow injection allows a continuous glow as long as the molecules and activating oxidants are pumped into the microfluidic chip.

We report the first experimental evidence of the enhancement of the chemiluminescence intensity of luminol by the introduction of metal nanoparticles in a microfluidic chip. Enhanced chemiluminescence intensity of luminol reacted with a weak oxidant, such as silver nitrate (AgNO_3_), in a microfluidic chip has been observed under catalysis (i.e., speed up of a chemical reaction) by gold nanoparticles^13^. It was demonstrated that smaller nanoparticles (11 nm in diameter) give a stronger chemiluminescence signal than larger ones (25 nm and 38 nm in diameter). To minimize the catalytic effect, we used nanoparticles 20 nm in diameter and larger.

## Materials and methods

**Figure 2:**
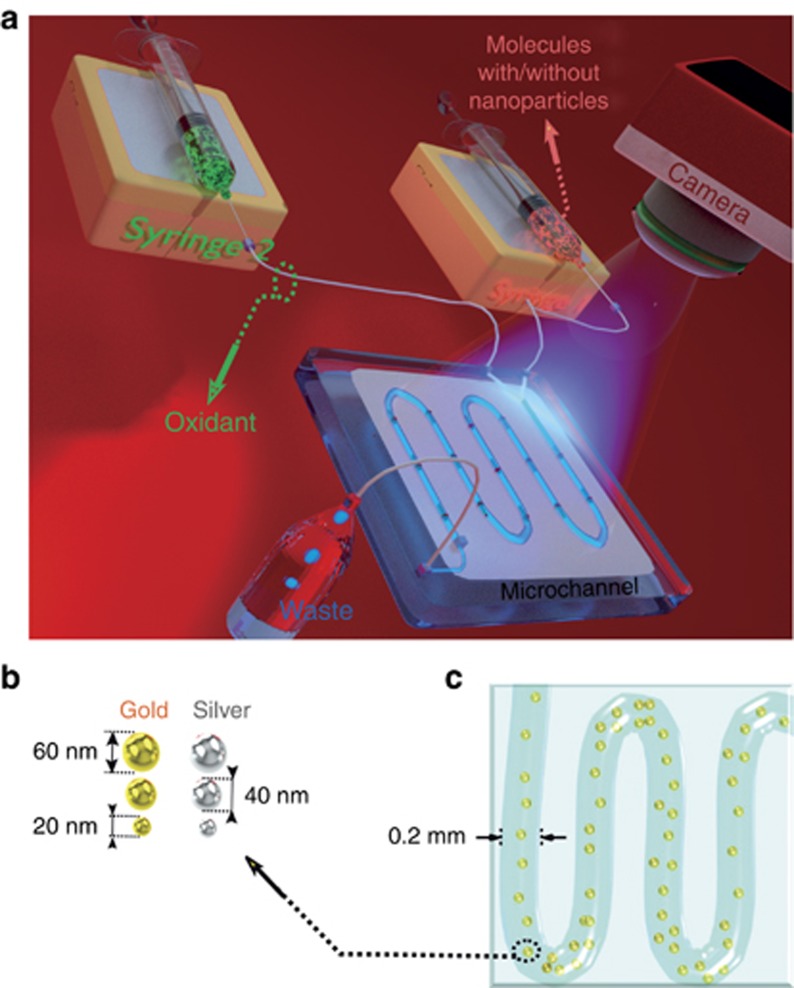
(**a**) Schematic of the system: microfluidic device with syringes that pump fluids through the serpentine on a PDMS microchannel on a chip. Organic waste is discarded through the outlet liquid reservoir. The emitted chemiluminescence signal is detected by the CCD. (**b**) Spherical gold and silver nanoparticles of *r*=10, 20 and 30 nm used in flow injection analysis. (**c**) Artistic impression of laminar flow of nanoparticles injected in microfluidic channel with 200 μm thick arms.

We have designed and fabricated a reusable microflow device with a serpentine channel 600 μm in width, 200 μm in depth and 600 μm in length, formed in polydimethylsiloxane (PDMS). A diluted oxidant, sodium hypochlorite (NaOCl), was injected into one part of the flow, and diluted luminol molecules were introduced into its other part. As a reference, we have been using 0.4 g of luminol with 50 mg of NaOCl bleach and 4 g of the oxidant sodium hydroxide (NaOH) for 1950 ml of water; for the sample, we have been using 0.2 g of luminol with 50 mg of NaOCl bleach and 2 g of oxidant NaOH for 1950 ml of water together with 50 ml of nanoparticles. The luminol solution was prepared either with or without nanospheres. The intensity of emitted light was detected by a charge-coupled device (CCD), Lumenera Infinity 2-3C (Lumenera Corporation, 7 Capella Crt. Ottawa, Ontario, Canada). Figure 2 shows a schematic of the studied system: a microfluidic device with syringes 180 μm in diameter that pump the fluid through a PDMS serpentine microchannel. It includes two inlets and one outlet. Luminol, NaOH, deionized water and nanoparticles were pumped through the first inlet (Syringe 1 in Figure 2a), whereas NaOCl and water were injected through the second inlet (Syringe 2 in Figure 2a). The colloidal nanoparticles we used are precisely manufactured monodisperse gold and silver nanoparticles from BBI Solutions (Kent, UK) suspended in water. They are passivated with polyethylene glycol to prevent aggregation. NaOH and luminol are the constituent parts of the chemical reaction that generates light. Organic waste was discarded through the outlet liquid reservoir as shown in the schematic in Figure 2a. During fabrication, the PDMS channel was molded over the 3D printed device. The layout for the mold was designed using the CAD Autodesk inventor (Stockport, UK). After printing, the channels were sealed using oxygen plasma for 30 s. We analyzed the chemiluminescence spectra at different flow rates and different concentrations of luminol. The maximum chemiluminescence intensity was obtained at the flow rate of 0.35 μl s^−1^. At higher flow rates, the reagent consumption was also increased compared to the slower flow rates. The limit of detection for the experimental setup was determined using three standard deviations and 20 repeats of images for each individual point, obtaining <110 μg ml^−1^. The gold and silver nanoparticles investigated in this study had radii *r*=10, 20 and 30 nm, as shown in Figure 2b. An artistic impression of the channel with gold nanoparticles is shown in Figure 2c.

To achieve a better understanding of the effect of gold and silver nanospheres on the efficiency of chemiluminescence emission by luminol, we performed spectrally resolved transmission measurements in the frequency range from 405 to 645 nm using a Jasco V570 spectrophotometer (28600 Mary's Ct, Easton, MD, USA) at room temperature as well as measurements of the chemiluminescence of luminol triggered by metallic nanoparticles.

## Results and discussion

**Figure 3:**
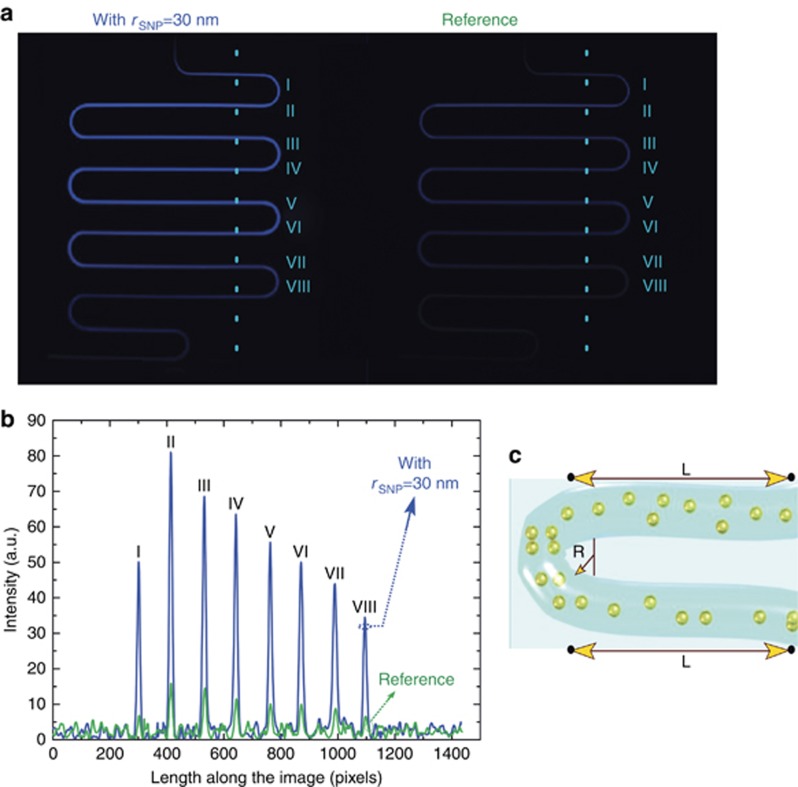
(**a**) Experimental images of the studied serpentine taken by a CCD camera. Luminol was injected with a flow rate of 0.35 μl s^−1^ with silver nanoparticles of *r*_SNP_=30 nm (left) to be compared with a reference signal detected in the absence of nanoparticles (right). (**b**) Intensity over a cross section of the images shown in **a**. Serpentine arms under investigation are labeled by roman numerals. Note: the subscript SNP denotes silver nanoparticles. (**c**) The trajectory of the particles or molecules between two points of the serpentine.

Here, we observe enhancement of the chemiluminescence intensity of a luminol flow in the presence of metallic nanospheres on a microfluidic chip. The experimental results captured by the CCD camera show a glowing serpentine channel in Figure 3a (left) compared to the barely seen serpentine channel in Figure 3a (right). In Figure 3a (left), syringe 1 shown in Figure 2a was filled up with luminol in the presence of silver nanospheres of radii *r*=30 nm. Figure 3a (right) shows a typical reference photograph: the imaged channel was filled with luminol without metal nanoantennas.

The serpentine arms in Figure 3a and the emission intensity along the serpentine arms in Figure 3b are designated by roman numerals. Figure 3b shows the change in the intensity of emission with the distance along the serpentine channel. The strongest enhancement occurs in arm II, which can be understood to indicate the best mixing between reagents in this arm and/or the most favorable distance between the light-emitting species and nanoantennas. The mixing in the microfluidic chip occurs based on the diffusion of particles from one laminar layer into the adjacent one. Efficient mixing occurs around the bends due to the Dean flow; therefore, arm II after the first bend shows the highest chemiluminescence intensity, and the efficiency of the Purcell effect increases. The remaining serpentine arms exhibit an exponential decrease in chemiluminescence intensity due to the chemiluminescence lifetime of the mixture. We have estimated the chemiluminescence lifetime of the mixture as a function of time, assuming an exponential decrease, in the serpentine arm schematically shown in Figure 3c. The flow rate of luminol in the channel is as follows:


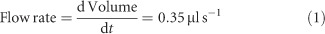


The cross section of the channel is *S*=*πR*^2^ = 3.14(100 μm)^2^ =3.14 × 10^−4^cm^2^.

The linear propagation velocity of luminol is Veloity_*x*_= 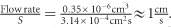


The characteristic length of the trajectory between two points along the serpentine channel, as shown in Figure 3, is 400 μm=4 × 10^−2^ cm, and thus the time elapsed between two neighboring points on the graph is: *t*=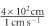 = 4 × 10^−2^s=40 ms.

**Figure 4:**
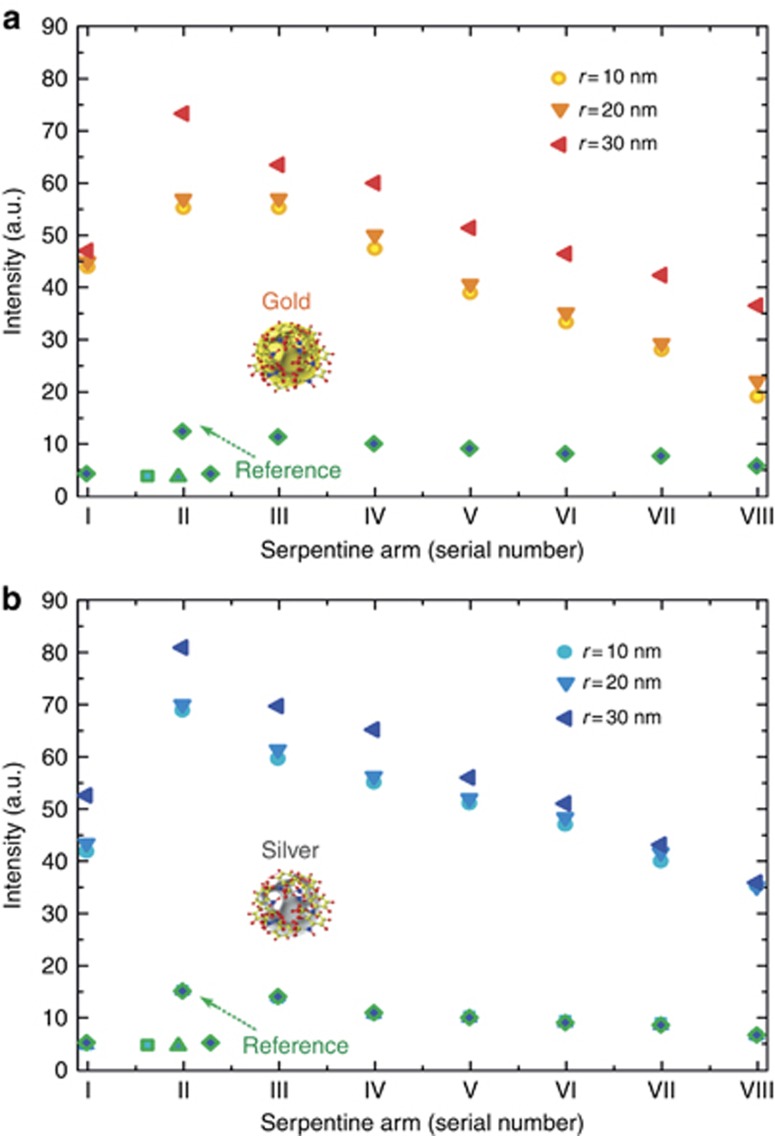
Chemiluminescence emission intensity of luminol in the serpentine arms for different radii of the nanospheres made of (**a**) gold and (**b**) silver compared to the intensity of emitted light from the reference sample (where water was injected instead of nanoparticles).

The experiments were repeated with injected gold and silver nanoparticles with radii *r*=10, 20 and 30 nm, as illustrated in Figure 4. The maximal enhancement was observed for gold or silver nanospheres with *r*=30 nm. Sun *et al*^14^ analytically described the photoluminescence enhancement as a result of the interplay of absorption and emission. It was shown^14^ that the increase in photoluminescence intensity due to the coupling to plasmonic modes is nonlinear with concentration when the particle size is unchanged. However, we did not observe any increase in the intensity of emitted light upon changing the flow rate (volume per unit time, μl s^−^^1^) of injected nanoparticles above 0.35 μl s^−^^1^. The maximum chemiluminescence intensity was obtained at flow rates of 0.35 μl s^−^^1^ and higher. The nonlinear increase in the intensity of chemiluminescence emission as a function of concentration and the independence of the emission intensity on the flow rate in a wide range of flow rates do not support the possible explanation of the enhanced chemiluminescence by the catalytic effect of metal. In our case, nanoparticles with ~*r*=30 nm provide the strongest enhancement of chemiluminescence. Figure 4a and 4b show the difference in the emission intensity between the different serpentine arms. The intensity in each arm was calculated by summing the pixels. The results suggest that an enhancement of up to ninefold occurs in arm II of the serpentine channel in the presence of silver nanospheres. Then, the signal exhibits an exponential decay. As shown in Table 1, however, the concentration of silver nanoparticles is one order of magnitude lower than the concentration of gold. One can conclude that silver nanoparticles induce a higher enhancement of chemiluminescence than gold nanoparticles. Therefore, the enhancement of luminol emission using silver nanospheres is stronger by a factor of up to 90 compared to using the same concentration of gold nanospheres.

**Figure 5:**
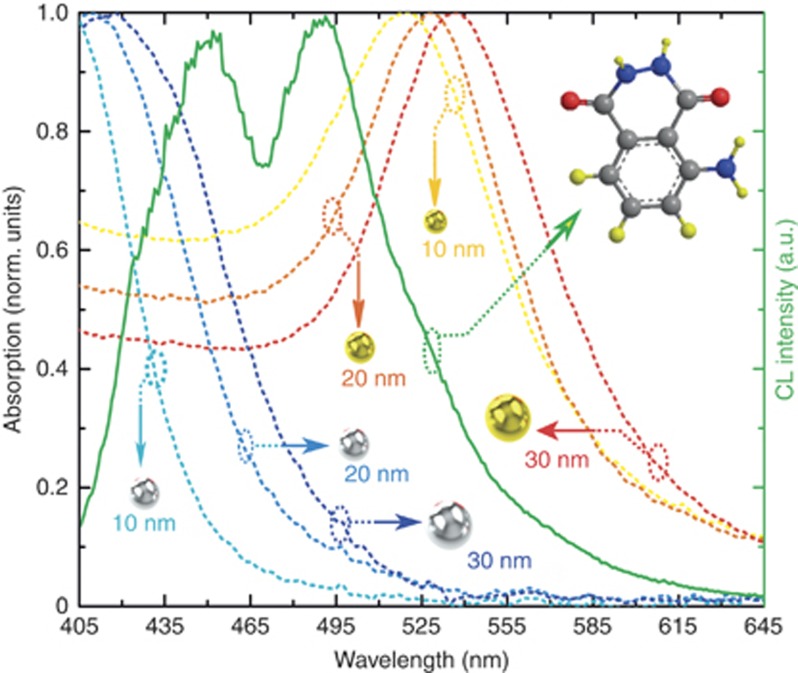
Overlap between measured chemiluminescence intensity of the luminol solution and the absorption spectra (dashed) of gold and silver nanoparticle acceptors in the original solution; the 3D structure of luminol is shown in the inset with a carbonyl double bond (C=O). All spectra have been normalized to their maximum values. For the emission measurement, we used 0.4 g of luminol with 50 mg of NaOCl bleach and 4 g of the oxidant NaOH for 1950 ml of water (original solution); for the absorption measurements, we used 0.2 g of luminol with 50 mg of NaOCl bleach and 2 g of oxidant NaOH for 1950 ml of water, together with 50 ml of nanoparticles.

We compare the transmission spectra of nanoparticles with the chemiluminescence emission intensity spectrum of luminol (Figure 5). The chemiluminescence spectrum of luminol exhibits two peaks at wavelengths of 452 and 489 nm that correspond to the emission of excited aminophthalate ions either bound to water molecules or unbound. Both types of ions are products of the oxidation of luminol. The molecular shape of luminol is shown by balls and sticks in the insets of Figure 5.

**Figure 6:**
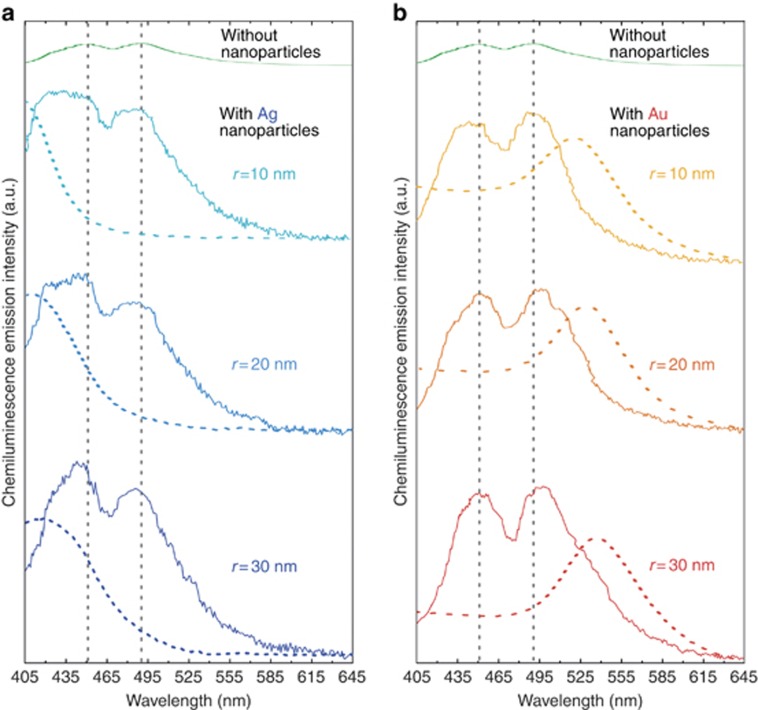
Measured chemiluminescence emission intensity controlled by (**a**) silver and (**b**) gold plasmonic nanoantennas. Chemiluminescence spectra of luminol molecule without nanoparticles (top) and with nanoparticles (below top). The dotted lines indicate the measured nanoparticles absorption spectra. For measurement without nanoparticles, we used 0.4 g of luminol with 50 mg of NaOCl bleach and 4 g of the oxidant NaOH for 1950 ml of water; for measurement with nanoparticles, we used 0.2 g of luminol with 50 mg of NaOCl bleach and 2 g of the oxidant NaOH for 1950 ml of water together with 50 ml of nanoparticles.

Figure 5 shows a significant overlap^15^ between the absorption spectra of the acceptor^16^ metal nanospheres studied here and the chemiluminescence intensity spectrum of the donor luminol. This overlap suggests that the enhancement of chemiluminescence here could be induced by the modification of the optical density of states in metallic nanoparticles in the vicinity of surface plasmon resonances^17, 18^. Notably, luminol emits at 452 and 489 nm (Figure 5), wavelengths that can be reabsorbed by the nanosphere silver surface plasmon situated below 450 nm and the nanosphere gold surface plasmon situated above 500 nm, respectively. This situation is favorable for the Purcell enhancement^19^ of the radiative emission rate mediated by plasmonic antennas. From Figure 6, the chemiluminescence emission peaks show spectral overlap with the resonance absorption of the nanoparticles. Thus, the effect of resonant light emission^20^ enhancement, as shown in Figure 4, could be linked to the optical coupling between luminol molecules and nanoparticles, just as a radio-antenna enhances radio emission^21^.

The chemiluminescence intensity of luminol in the presence and in the absence of nanoparticles is shown in Figure 6a (silver) and 6b (gold). The peaks of emission intensity at 452 and 489 nm are indicated by dashed lines. The presence of plasmonic nanoparticles changes the chemiluminescence characteristics of luminol, leading to a multi-fold intensity enhancement as well as to the strong spectral modification of the chemiluminescence emission peaks. For instance, for silver nanoparticles with *r*=30 nm, the chemiluminescence peak is enhanced, and the emission peak of the luminol at 452 nm is modified. Here, the resonant absorption peak of silver nanoparticles overlaps substantially with the luminol emission peak at 452 nm (see the absorption spectrum shown by the blue dashed line). This overlap suggests that the enormous chemiluminescence enhancement results from the interaction between excited-state luminol and the ensemble of optical modes in the system. A source of light (a luminol molecule in our case) emits photons to a medium characterized by some given density of photonic states. If at the emission frequency this density is lower than in vacuum, the radiative efficiency is increased. In the presence of metallic nanoparticles, the density of photonic states increases resonantly at certain characteristic frequencies associated with the plasmon modes of metallic objects. If the emission band of luminol overlaps with the spectral region of the plasmon-induced increase in the photonic density of states in the medium, the radiative efficiency of luminol is enhanced. We attribute this result to the Purcell enhancement^19^ of the radiative recombination rate of luminol molecules. The Purcell factor *F*_p_ is governed by the overlap of the emission spectrum of luminol and the absorption band of metallic nanoparticles. The most efficient Purcell enhancement of the radiative recombination is observed if the emission wavelength is resonant with the plasmon absorption peak in the antennas^22^. Such coupling between the emitter and the nanoparticle surface plasmon must be sensitive to the particle material (gold or silver), the particle size and the spacing between the emitting luminol molecule and the nearest nanoparticle. If the plasmon resonance in a nanoparticle is blue-shifted due to the smaller radius of the particle, the chemiluminescence spectrum is weakened and broadened near 452 nm. In the case of gold nanoparticles, by reducing the radius of the particle, the chemiluminescence spectrum is weakened and sharpened near 489 nm. In all cases, the chemiluminescence emission spectrum is shifted from its original position towards the nanoparticle wavelength corresponding to the resonant absorption of the particle. In addition, the presence of a strong interactor, metal, in the microfluidic chip could induce the shift in the emission spectrum of luminol.

**Figure 7:**
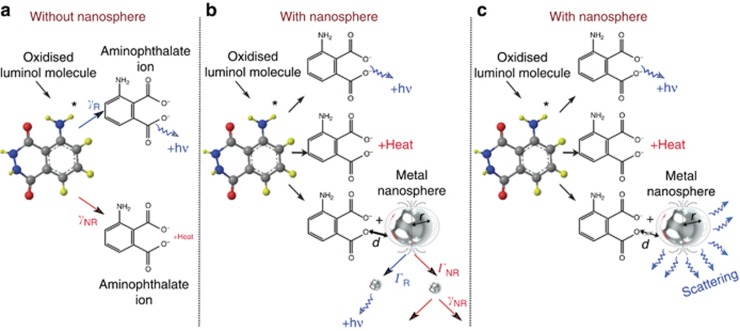
Schematics of enhancement mechanisms of chemiluminescence: (**a**) an oxidized luminol molecule can convert to an aminophthalate ion and a photon or heat; or (**b**) an oxidized luminol molecule can convert to an aminopthalate ion and an emitted photon, to an aminophthalate ion and heat, or to an aminophthalate ion within a distance *d* from the nanosphere of radius *r*, enhancing the chemiluminescence intensity. (**c**) Far-field scattering of light emitted by luminol molecules with randomly illuminated metallic nanospheres^23^.

The enhancement mechanisms of chemiluminescence emission are illustrated in Figure 7^23^. In the case of luminol, radiative recombination could compete with non-radiative decay processes^24^. There is little light emitted in the course of a chemiluminescence reaction because the probability of emitting a photon is much lower than the probability of decaying through a non-radiative channel (e.g., by collisions or resonant energy transfer to another molecule). This case is the typical one where nanoantennas are useful^25^. Indeed, they can introduce a fast decay channel for the luminophore that can compete with the non-radiative processes, which are otherwise much faster than photon emission processes. In the presence of metallic nanospheres, the very low quantum yield of luminol molecules, *η*_CL_ =0.001–0.1, could be replaced by the higher quantum yield of metallic particles, which depends on the radius and the material (gold or silver) of the nanosphere.

The glow of luminol is a manifestation of chemiluminescence: light emission resulting from an exothermic chemical reaction. Spontaneous emission can be enhanced by the presence of metal nanoparticles. Chemiluminescence can also be amplified by chemical catalysis^26^, e.g., by nanostructure-induced catalysis enhancement^27, 28^ or optically by using resonant structures, such as nanoantennas, quantum dot metamaterials^22^ or silver islands^29^. The overall efficiency of chemiluminescence, *η*, can be expressed as *η*=*η*_C_ *η*_e_*η*_F_, where *η*_C_ is the fraction of reacting molecules that may be excited, and *η*_e_ is the percentage of molecules that actually are excited. This value describes the efficiency of the energy transfer. Finally, *η* = 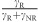 is the quantum yield of the emitter^7^, where *η*_F_ accounts for the probability of radiative decay for a single molecule, and *γ*_R_ and *γ*_NR_ are the radiative and non-radiative decay rates, respectively. Chemiluminescence is limited in efficiency because excited molecules can lose their energy through non-radiative processes, such as internal conversion and intersystem crossing. An excited molecule can decay either radiatively at the rate *γ*_R_ or non-radiatively at the rate *γ*_R_. These competing effects are schematically depicted in Figure 7a. In the presence of nanoantennas^30^ (Figure 7b), the initial population of excited molecules can decay radiatively at the rate *Γ*_R_ or non-radiatively at the rate *Γ*_R_. In the last case, the ratio of radiative and non-radiative decay rates can be changed. The non-radiative decay rate of luminol in the presence of nanoantennas is modified by non-radiative energy transfer to the antenna, as shown in Figure 7b. The chemiluminescence emission enhancement and spectral shape can also be modified by the far-field scattering mechanism schematically presented in Figure 7c. The quantitative description of the mechanism of chemiluminescence enhancement will be studied in our future work.

## Conclusions

In conclusion, we have demonstrated multi-fold enhancement of the chemiluminescence intensity during flow injection in a microfluidic chip. We have observed that pumping nanoparticles into a microfluidic device fabricated in PDMS prolongs the glow time of luminol. The enhancement of chemiluminescence by metallic nanoparticles may be due to the following factors: (i) the uniform intensity of chemiluminescence emission due to thorough mixing of the reagents and maximized intensity due to the location of emitters at distances that are favorable for interaction with the metal nanoparticles (antennas) and (ii) the collective response of conduction electrons in the metal. The optical field of plasmon modes is localized in the vicinity of the surfaces of metallic nanoparticles. As a result, the lightning antenna effect exhibits resonant amplification if the excitation frequency coincides with a localized surface plasmon resonance of the particle. We have proposed two possible mechanisms for chemiluminescence enhancement during flow injection. (i) The rate of emission by a chemophore emitter can be enhanced by an antenna effect. The Purcell effect may be responsible for the amplification of the radiative decay rate due to the enhanced density of optical states accessible for decay of the molecular excitation. (ii) The observed enhancement and modification of the spectral shape could also be due to far-field scattering. The observed chemiluminescence enhancement provides the first demonstration of a resonant enhancement of luminol flow in the presence of nanoparticles. From a technological perspective, the use of microfluidic devices (i) reduces the luminol consumption, (ii) controls mixing and (iii) controls the concentration of nanoparticles. This innovation is important for achieving uniform emission intensity and the favorable location of emitting species with respect to the nanoparticles. We have integrated the chemiluminescence analysis setup on a chip (known as lab-on-a-chip) to facilitate imaging. Our observations indicate that noble metal nanoparticles can readily be used to enhance chemiluminescence even in microvolume samples. This observation is an essential step toward developing microfluidic chips with gain media and toward improving the detection limits of chemiluminescence.

## Figures and Tables

**Table 1:** Concentrations of gold and silver nanoparticles

***r***	**SNP**	**GNP**
10	7 × 10^10^	7 × 10^11^
20	9 × 10^9^	9 × 10^10^
30	2.6 × 10^9^	2.6 × 10^10^

*r* is the radius of the nanoparticle in nm. GNP is an abbreviation for gold nanoparticles in nanoparticles in particles per ml (from nanoparticle manufacturer BBInternational).
